# Appropriateness of carotid plaque and intima-media thickness assessment in routine clinical practice

**DOI:** 10.1186/1476-7120-6-52

**Published:** 2008-10-16

**Authors:** Liz Andréa Villela Baroncini, Aguinaldo de Oliveira, Enrique Antonio Vidal, Graciliano José França, Paulo Sérgio Dalla Bona Stahlke, Alexandre Alessi, Dalton Bertolim Précoma

**Affiliations:** 1 Center of Health and Biological Sciences-Pontifical Catholic University of Paraná, Paraná, Brazil; 2 Federal University of Paraná, School of Medicine, Department of Surgery, Paraná, Brazil

## Abstract

**Objectives:**

To describe the findings and evaluate appropriateness of a carotid artery study including the measurement of IMT, the presence of atherosclerotic plaque, and their correlation with cardiovascular risk factors.

**Methods:**

555 patients (220 men; 67.06 ± 12.44 years) were included in the study. 120 patients (21.62%) presented carotid plaque: 108 (19.45%) in patients with at least one risk factor and 12 (2.1%) in patients without risk factors. With respect to appropriateness of the present studies: 65% were appropriate, 22% were uncertain and 13% were inappropriate. The IMT medians were higher in males (0.0280; 95% CI, 00.82 to 0.478; *p *= 0.0057) and in hypertensive patients (0.391; 95% CI, 0.0190 to 0.0592; *p *= 0,001). There was a linear increase in mean IMT for each year increased in age (0.0059; 95% CI; 0.0050 to 0.0067). Carotid plaque was more frequent in patients with CAD (*p *= 0.0002), diabetes (*p *= 0.024) and hypertension (*p *= 0.036).

**Conclusion:**

Assessment of carotid arteries identified increased incidence of plaque in patients with CAD, diabetes and hypertension. IMT was increased in older patients, hypertensive patients and males. Forty-five percent of the patients were studied based on uncertain and inappropriate reasons.

## Introduction

It has been proven that the atherosclerotic changes in the carotid artery mirror general atherosclerosis. Ultrasound measurements of IMT and plaque occurrence in the carotid arteries are important not only for the assessment of structural alterations but also because the extent of atherosclerosis in these vessels reflects the severity of arterial damage in other vascular territories [1]. Epidemiological studies and clinical trials have shown that carotid artery intima-media thickness can identify prevalent and incident cardiovascular disease (CVD) events, and progression and regression of atherosclerosis [2]. Intima-media thickness (IMT) of the carotid arteries is increasingly used in clinical trials as an important risk marker to investigate normal aging and preclinical atherosclerosis [3]. Several studies demonstrated that carotid IMT is significantly associated with risk for myocardial infarction, stroke, death from coronary artery disease, or a combination of these events [4]. Interestingly, in daily clinical practice, with the diffusion of knowledge and the education of public opinion, many physicians request carotid ultrasound studies even in patients without cardiovascular risk. We carried out this study to describe the findings and evaluate the appropriateness of carotid artery studies with ultrasound, including the measurement of ITM; the presence or not of atherosclerotic plaque; and their correlation with traditional cardiovascular risk factors in routine exams from our Doppler vascular laboratory.

## Methods

### Patients

Five hundred eight patients (229 men and 351 women; mean age 67.06 ± 12.44 years) who underwent a carotid artery ultrasound in our Doppler vascular laboratory were screened for carotid plaque and IMT measurement. Information on demographic characteristics and risk factors were collected using a structured questionnaire. Indication to perform the exam was recorded based on the referring physician's request. In addition, patients were asked about the indication for the exam and the presence or not of hypertension, diabetes mellitus, dyslipidemia, coronary artery disease, stroke and current smoking. Hypertension was defined by a history of treated hypertension. Smoking history was coded as never or current smoker. Subjects were classified as diabetics when treated for insulin-dependent or non-insulin-dependent diabetes. The use of lipid-lowering drugs was assessed and the history of myocardial infarction, angioplasty, or coronary artery by-pass surgery was recorded. A positive coronary artery disease (CAD) history was defined by the presence of any of these diseases. History of stroke or transient ischemic attacks was obtained for all cases. In addition, we classified the studies according to three categories: appropriate, inappropriate and uncertain. Appropriate studies were those for which patients had one or more risk factors; uncertain studies were those for which patients had nonspecific symptoms; and the studies for which patients had no risk factors or symptoms were considered to be inappropriate. The patient's baseline characteristics and the indications for carotid ultrasound based on medical request or patient's information are displayed in Tables 1 and 2.

**Table 1:** Patient's baseline characteristics

**Patients with risk factors (N/%)**	**451 (81.26%)**	**Carotid plaque**
Sex (M/F)	175/276	
Age (y ± SD)	67.6 ± 11.7	
All patients		108 (19.45%)
History of hypertension (N/%)	333 (73.8%)	
History of dyslipidemia (N/%)	249 (55.2%)	
History of diabetes mellitus (N/%)	100 (22.2%)	
Cardiovascular history (N/%)	71 (15.7%)	
Current smoking (N/%)	65 (14.4%)	
***Patients without risk factors (N/%)***	***104 (18.73%)***	***12 (2.1%)***
Sex (M/F)	42/62	
Age (y ± SD)	64.6 ± 15.1	

**Table 2:** Indications for carotid ultrasound study (N/%)

**Patients with risk factors**	**451 (81.26%)**
Check-up	238 (51.8%)
Dizziness or vertigo	60 (13.1%)
Syncope	35 (7.6%)
Stroke	30 (6.5%)
Preoperatory evaluation	16 (3.8%)
Transient amnesia or disorientation	14 (3.1%)
Previous carotid artery disease	9 (1.9%)
Unspecific symptoms	56 (12.2%)
***Patients without risk factors***	***104 (18.73%)***
Check-up	72 (71.2%)
Dizziness or vertigo	11 (10.8%)
Syncope	3 (3.1%)
Stroke	5 (5.4%)
Preoperatory evaluation	1 (1%)
Transient amnesia or disorientation	1 (1%)
Previous carotid artery disease	2 (2.1%)
Unspecific symptoms	6 (6.1%)

### Ultrasound measurements

Measurements were made with a high-resolution B-mode ultrasonography (Philips Medical Systems' HD11 platform) with a broadband width linear array transducer L 3–12 MHz. Sonography and readings were carried out by trained and certified sonographers. Measurements of IMT were take on the distal 10 mm of both right and left common carotid artery in the far wall. Five determinations were conducted on each side and the average measurement was used for the IMT. An artery was classified as being affected by plaque if there was a localized thickening > 1.5 mm that did not uniformly involve the whole left or right carotid artery with or without flow disturbance. The vascular ultrasonographist identified plaques on common, internal and external carotids at the time of ultrasound measurement.

### Statistical analysis

Categorical variables were expressed as percentages and continuous variables were expressed as mean ± SD, with a 95% confidence interval (CI) and a significance level of 5%. Multiple linear regression analysis was used to assess the association between cardiovascular risk factors and IMT. Age and sex were used as control variables. Twenty-five IMT measurements identified as possible outliers or influential cases were eliminated using the DFFITS procedure. χ^2 ^test with Yates correction was used to correlate each risk factor and the presence of plaque. The regression logistic model was used to assess all variables with respect to the presence of plaque. Statistical significance was indicated by a value of *p *< 0.05. Analyses were performed using Statistica/W version 5.1 (StatSoft, Tulsa, Okla.).

## Results

**Figure 1:**
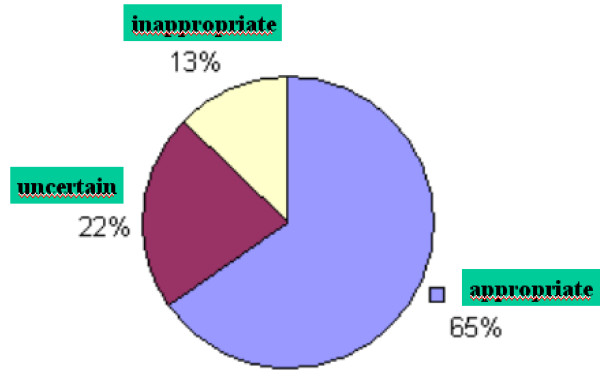
Appropriateness graphic.

**Figure 2:**
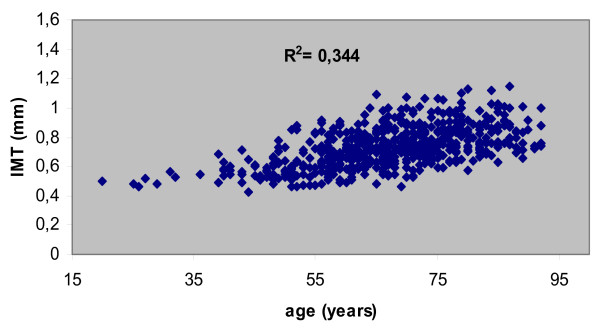
Dispersion diagram between IMT and age (0.0059; 95% CI,0.0050 to 0.0067).

A total of 555 patients were analyzed. Four hundred fifty-one (178 men; 67.6 ± 11.7 years) had at least one risk factor. One hundred four patients (42 men; 64.6 ± 15.1 years) had no risk factor. The appropriateness of the studies is displayed on figure 1. Risk stratification or "check-up" was the main indication for carotid ultrasound study in most patients (55.85%) even in subjects with no risk factor at all, followed by dizziness or vertigo (12.79%). Hypertension was present in 73.8%; dyslipidemia in 55.2%; diabetes in 22.2%; coronary artery disease (CAD) in 15.7%; and current smoking in 14.4%. One hundred twenty patients (21.62%) had carotid plaque: 108 (19.45%) in patients with at least one risk factor and 12 (2.1%) in patients without risk factors. (Tables 1 and 2). The IMT medians were higher in males (0.0280; 95% CI, 00.82 to 0.478; *p *= 0.0057) and in patients with hypertension (0.391; 95% CI, 0.0190 to 0.0592; *p *= 0.001). There was a linear increase in mean IMT for each year increased in age (0.0059; 95% CI; 0.0050 to 0.0067). (Table 3). (Figure 2). Other risk factors did not influence IMT measurements. Plaque occurrence tended to influence IMT measurement (*p *= 0.067). Plaque was more present in patients with CAD (*p *= 0.0002), with diabetes (*p *= 0.024), and with hypertension (*p *= 0.036). There was no significant difference in patients with dyslipidemia (*p *= 0.158) or current smokers (*p *= 0.766). (Data not shown).

**Table 3:** Ultrasound measurements (95% CI)

**Variable**	**Controls**	**P value**	**Lower limit**	**Upper limit**
Sex	0.280	***0.0057***	0.0082	0.0478
Age	0.0059	***< 0.001***	0.0050	0.0067
Hypertension	0.0391	***< 0.001***	0.0190	0.0592
Diabetes	0.0103	0.4251	0.0358	0.0151
Current smoking	0.0095	0.5293	0.0201	0.0390
Dyslipidemia	0.0081	0.4109	0.0273	0.0112
CAD	0.0136	0.3636	0.0430	0.0158

## Discussion

**Figure 3:**
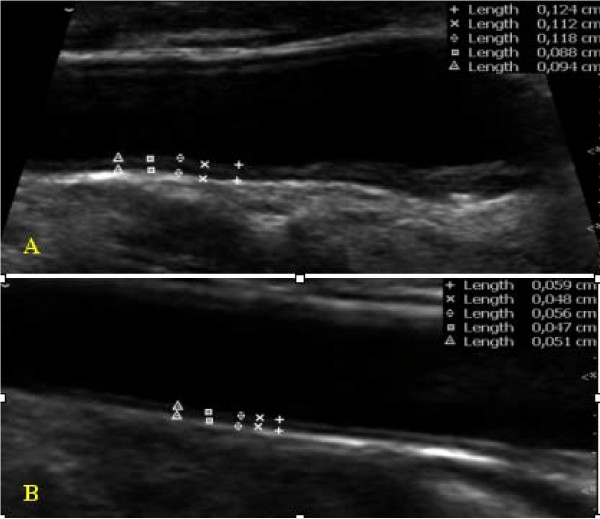
**Carotid intima media thickness.** A Increased and irregular IMT. B Normal IMT.

The Mannhein Carotid IMT Consensus [3,5] and the ASE Consensus Statement [4] recommended IMT measurements for patients at intermediate CVD risk and in subjects with the following clinical circumstances: (1) family history of premature CVD in first-degree relatives; (2) individuals younger than 60 years old with severe abnormalities in a single risk factor who otherwise would not be candidates for pharmacotherapy; (3) women younger than 60 years of age with at least two CVD risk factors; and (4) in all epidemiological and interventional trials dealing with vascular diseases to better characterize the population investigated. However, carotid ultrasound and the assessment of IMT are highly requested today by most physicians, in particular by cardiologists. In the present study, the "check-up" was the main indication for carotid ultrasound study, followed by dizziness or vertigo, even in patients with no risk factors. The risk stratification of an individual patient without clinically apparent atherosclerosis (primary prevention) is oftentimes complex. There are patent scenarios in which an individual is judged to be at low risk of future CV events by traditional risk stratification scoring, however, because of a young age, the presence of a strikingly abnormal single risk factor, or of an emerging risk factor, the incremental information provided by a carotid IMT may more accurately assess this risk [6]. The ASE Consensus Statement also recommends the use of a semi-automated border detection program with validated accuracy for assessment of IMT [4], but since most vascular labs do not have an automated system to determine IMT and vascular age, the sonographers regularly use manual reading techniques. In vascular laboratory daily routine, the ultrasonographist easily visualizes a normal and an irregular intima-media layer in carotid vessels or atherosclerotic plaque. (Figure 3). Porter et al [7] showed that dynamic range, gain and probe distance significantly alter lumen diameter and IMT measurements made even using image analysis software. In the present study, we made 5 consecutive measurements for IMT in each common carotid artery in order to minimize the errors. Interestingly, we found atherosclerotic plaque in only 19.45% of patients with at least one risk factor and in only 2.1% of patients without any risk factor. It is clear that the prognostic performance of a method is heavily related to the type of population. The present study included not only patients indicated by a cardiologist but also the general population, referred by other medical specialities such as homeopathic medicine, gerontology and internal medicine, thus including some patients with no cardiovascular risk at all. We also considered cardiac and cerebrovascular events. As in previous studies [1,8,9], we found higher values of IMT in hypertensive subjects, however with respect to other risk factors, we did not find any influence on this measurement. The progression of atherosclerotic disease may occur at different rates and as a result of different risk factors in each vascular bed [10]. We also found higher values of IMT in males and older patients. In view of all these findings, some questions arise, for example: 1 – What information should we give to the referred physician?; 2 – What will they do in a totally asymptomatic patient with no risk factor? Ultrasonographists, therefore, should be precise in their report regarding the presence or not of plaque and the measurement of IMT. These findings could change risk stratification for asymptomatic subjects and their primary physician should be aware of this possibility. In addition, carotid IMT has proven to be a powerful tool for assessing cardiovascular risk. Atherosclerosis is not a single disease entity, but a process consisting of the responses to numerous insults to the endothelium and smooth muscle cells of the arterial wall. This "response-injury" hypothesis is formulated by numerous observations in humans and animals, and the entire process from the earliest recognizable lesion (fatty streak) to advanced lesions in atherosclerosis (fibrous plaques) is tightly linked to an inflammatory response [7,11-18]. If, in a patient with no risk factor, we find an increased IMT or a small carotid plaque the personal physician should most likely inquire about inflammatory markers. Finally, there are several studies [19-21] evaluating the prognoses of increased IMT and raise the question of whether or not one should treat this condition. We hope to soon understand the significance of an increased IMT or the presence of small plaque in healthy subjects.

## Conclusion

In a routine vascular laboratory the assessment of carotid arteries identified increased incidence of plaque in patients with CAD, diabetes and hypertension. In addition, carotid IMT was increased in hypertensive patients and males. Older patients presented higher values of carotid IMT. Forty-five percent of the studies were categorized as uncertain and inappropriate.

## Competing interests

The authors declare that they have no competing interests.

## Authors' contributions

LAVB participated in the study design, wrote and oriented the manuscript. AO participated in data collection. EAV participated in data collection. GJF participated in data collection. PSDS participated in data collection. AA participated in the study design. DBP participated in the study design. All authors read and approved the final manuscript.
